# Effects of food availability on larval development during ontogenetic niche shift in a marine annelid

**DOI:** 10.1098/rsob.250135

**Published:** 2025-10-08

**Authors:** Nadine Randel

**Affiliations:** ^1^ Department of Physiology, Development, and Neuroscience, University of Cambridge, Cambridge, UK; ^2^ Deprtment of Zoology, University of Cambridge, Cambridge, UK

**Keywords:** larval development, ontogenetic niche shift, resource allocation, *Platynereis*

## Abstract

Many marine invertebrates have a biphasic life cycle with a free-swimming larva and a bottom-dwelling adult. The transition from a planktonic to a benthic lifestyle is a significant step in the animal’s life history, highly regulated and influenced by external and internal factors. Since the readiness to settle and the presence of a suitable seafloor habitat do not always coincide, larvae sometimes need to extend their planktonic phase. Little is currently known regarding how larvae partition their energy for coordinating development and growth according to food type and availability in their settlement habitat. Here, I investigate the effect of food availability and type on development in *Platynereis dumerilii* larvae. I assessed cell proliferation, growth and feeding onset over six days using two different food sources. The results indicate that food availability and type affect larval growth, with starved larvae slowing development and conserving resources, whereas fed larvae allocate resources to brain development and posterior growth. Overall, this work contributes to our understanding of how competent marine larvae regulate the duration of their planktonic phase and how nutritional status affects development.

**Figure:**
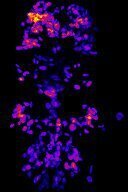

## 1. Introduction

Every animal is adapted to its ecological niche and reacts to its environment. However, many animals change their niche during their lifetime. This can be as simple as an increase in body size, necessitating different food sources and shelters [1]. The transition between different niches and the resulting reorganization of functional systems (e.g. nervous system and locomotor system) has been termed ontogenetic niche shift [2]. Ontogenetic niche shift occurs in many invertebrate and vertebrate taxa such as insects, molluscs, annelids, echinoderms, fishes and amphibians [3,4]. It is an important step in an animal’s lifetime, essential for gaining sexual maturity and preserving ecological communities [5–9]. In addition, the transition of planktonic marine larvae to bottom-dwelling adults has come into focus due to its significance for marine ecosystem conservation, fisheries, aquaculture and biofouling [10–13].

A hallmark of these marine invertebrate taxa is the biphasic life cycle. The free-swimming planktonic larvae inhabit the water column from a few hours to several months before they settle down to the seafloor [14]. Feeding larvae normally spend longer in the plankton, whereas a short planktonic phase is often associated with lecithotrophy, where larvae obtain their nourishment from maternally provided yolk or lipid droplets.

By the end of the larval stage, the larva has developed competency, the ability to respond to environmental cues and settle onto the seafloor [15–20].

Due to the sometimes highly specific nature of environmental cues required to induce settlement, competent larvae might not be able to settle. As a result, they either spontaneously metamorphose, die within a few days or survive an extended period in the plankton but lose the ability to develop into adults [14]. Consequently, the ability to maintain competency is important for the animal’s fitness.

Prior studies have shown that starved planktotrophic larvae can prolong their larval phase [21–23]. However, little is known about the ability to maintain competency in planktotrophic and lecithotrophic larvae [21]. Additionally, lecithotrophic larvae, feeding on maternally provided yolk reserves, may be incapable of feeding, while others are facultative planktotrophic, feeding on provided yolk and having the capability to feed on plankton, or, as in the marine annelid *Platynereis dumerilii* (RRID:NCBITaxon_6358) they settle as pre-metamorphic hemisessile feeding larvae [24–28]. For example, competent planktonic *Platynereis* larvae start probing the seabed for food before settlement [29].

**Figure 1.**
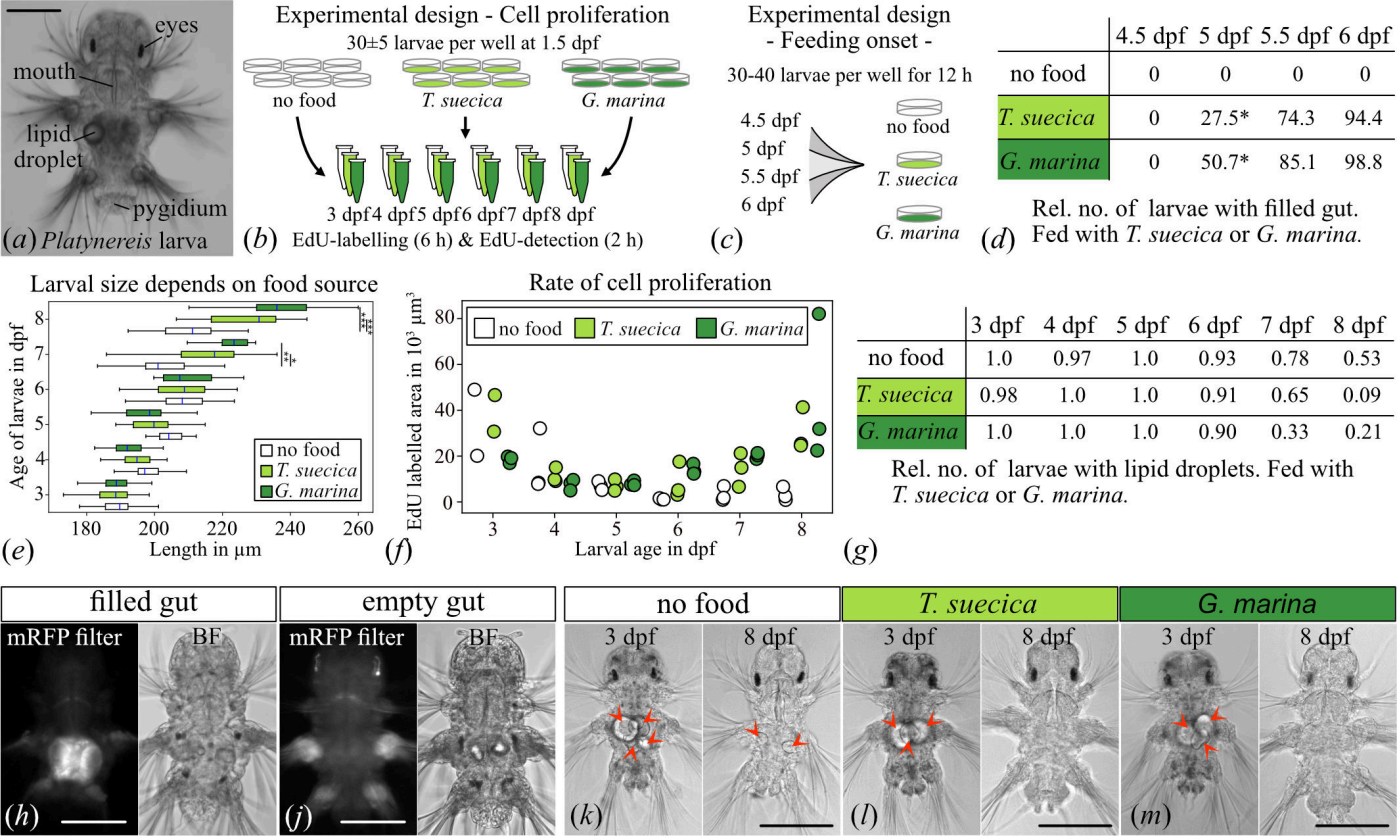
Effect of microalgae on larval size, cell proliferation and feeding behaviour during ontogenetic niche shift. (*a*) Light micrograph of a 6 dpf old *Platynereis* larva. (*b*) Experimental design of the cell proliferation assay and (*c*) testing of feeding onset. (*d*) Proportion of larvae with microalgae in the gut (*p ≤ 0.05). (*e*) Box and whisker plots of larval size across ages and food conditions (*p ≤ 0.05, **p ≤ 0.001 and ***p ≤ 0.0001). (*f*) Total area of EdU-labelled cells from three larvae per age and food conditions. (*g*) Proportion of larvae with 1−4 lipid droplets, feeding on *T. suecica* and *G. marina*. (*h–j*) Light and fluorescence micrograph of *T. suecica* fed and unfed larvae, imaged with red fluorescent protein (RFP) filter. (*k–m*) Light micrograph of 3 and 8 dpf old larvae with maternally provided lipid droplets (arrowheads), under different food conditions. (*d*,*e*,*g*) Statistics: paired *t*‐test, Holm–Bonferroni correction. Scale bar, 100 µm.

Despite the importance of maintaining larval competency, our understanding of the underlying mechanisms remains limited. Although prior studies have highlighted the role of food in many species, its effects on development remain poorly understood. This study investigates how food availability affects the development of competent *P. dumerilii* larvae. *Platynereis* is a well-established model with a biphasic life cycle, featuring a lecithotrophic larva that develops into a bottom-dwelling adult (figure 1*a*) [24]. After hatching, the gut of the larva is not fully formed, and four maternally provided lipid droplets serve as an initial energy source. Later they feed on various species of green algae and diatoms on the seafloor, including the microalgal species used in this study: the unicellular, free-swimming green alga *Tetraselmis suecica* and the benthic, biofilm-forming diatom *Grammatophora marina*.

My work adds insights into how food availability affects the timing of feeding onset, use of the maternally provided lipid droplets and larval growth rates.

## 2. Material and methods

### 2.1. *Platynereis dumerilii* and microalgal culture

Animals and microalgae are cultured in the Marine Invertebrate Culture Unit at the University of Exeter, UK and were provided by the laboratory of Dr Elizabeth Williams [29].

### 2.2. Diatom biofilm preparation

To enhance the biofilm formation, 6-well plates (ThermoFisher Scientific 130184 bioLite 6-well multidish) were coated with poly-D-lysine (Sigma-Aldrich P6407, final concentration 5 µg ml^−1^) for 2 h at 37°C and subsequently dried at 50°C. The coated 6-well plates were then incubated for 36 h with 3 ml *G. marina* diatom culture medium. The medium consisted of filtered seawater supplemented with F/2 nutrient medium (1 ml per 2.5 l; ZM Systems), and a silicate solution (1 ml per 2.5 l; ZM Systems). Afterwards, the 6-well plates were gently washed and covered with 1 µm-filtered UV-light-sterilized artificial sea water (Tropic Marin Pro-Reef salt) at a salinity of 33 ppt. To grow the diatom biofilm, 3 ml of *G. marina* cultivated in the diatom culture medium was added to the prepared 6-well plates, ensuring food was not a limiting factor.

The biofilm coverage was calculated using Biofilm_coverage_macro.ijm [29]. It was estimated at 6–12% (electronic supplementary material, S1).

### 2.3. Experimental design for cell proliferation assay

From two batches of larvae, 30 ± 5 larvae with differing parentage were added to the prepared 6-well plates, with three biological replicates. The 1.5 day post fertilization (dpf) larvae were either raised on *G. marina* biofilm or fed with 50 µl *T. suecica* algal culture. The larvae were kept in an 18°C incubator with 18 h light : 6 h dark photoperiod.

### 2.4. Cell proliferation assay and DAPI staining

Newly synthesized DNA was labelled in whole larvae and detected using a Click-IT EdU (5-ethynyl-2′-deoxyuridine) Alexa Fluor 647 Imaging Kit (Invitrogen C10340) according to the kit instructions. *Platynereis* larvae were incubated with EDU at 3, 4, 5, 6, 7 and 8 dpf for 6 h. Afterwards, the larvae were fixed for 1 h with 4% PFA (paraformaldehyde solution 16% (Agar Scientific AGR1026)) in PBW (phosphate buffer (PBS) + 0.1% Tween20 (Sigma-Aldrich P9416)) and washed in PBW. After a final washing step with PBS, the larvae were incubated for 2 h with the reaction cocktail (EdU kit) and washed in PBS. Finally, larvae were stained with DAPI (4′,6-diamidino-2-phenylindole) (0.5 µmol ml^−1^) (Merck D9542-1mg) for 10 min, and stored in 2,2-thiodiethanol (Merck 166782-500g) at 4°C in the dark until imaging. Sample size per age and condition was 12−51 larvae (figure 1*b*).

### 2.5. Experimental design for feeding onset characterization

About 30–40 larvae from two batches were incubated in 6-well plates with *G. marina* biofilm, an excess of *T. suecica* algal culture, or no food (three biological replicates) at 4.5, 5, 5.5 and 6 dpf for 12 h (figure 1*c*). After fixation with 4% PFA in PBS for 1 h, the larvae were washed with PBS and imaged within two weeks.

### 2.6. Imaging

#### 2.6.1. Confocal microscopy imaging of EdU-labelled larvae

Larvae were mounted in 2,2-thiodiethanol and imaged with a Leica upright SP5 confocal microscope (40× oil objective, NA 1.25) in the imaging facility at the University of Cambridge (UK), Department of Zoology.

#### 2.6.2. Bright field microscopy imaging of larval size, lipid droplets and gut content

Larvae were imaged with an Axioscope 40 (20× air objective) and recorded with Micromanager 2.0 in the imaging facility at the University of Cambridge (UK), Department of Zoology. The microalgal autofluorescence was recorded with 20% light intensity and 200 ms exposure time, using an RFP filter.

Length of the EdU-labelled larvae was measured from the head to the pygidium using Fiji [30]. In addition, the presence or absence of maternally provided lipid droplets was recorded for each larva. According to the experimental set-up, incubation time with food in the EdU-labelled larvae was 36–156 h.

### 2.7. Data analysis

Data analyses of larval size, lipid droplets and gut content were performed using Python 3.7, and estimation of EdU-labelled area was done using an ImageJ (RRID:SCR_003070) Macro. All scripts are available at commit nrandel_statistics_d64cdf3_2025-04-29 (29 Apr 2025) on GitHub: https://github.com/nrandel/statistics/tree/d64cdf3f42ae89fd44b8aa73adeab66e758e0615/Randel_2025.

#### 2.7.1. Estimation of EdU-labelled area

Each three-dimensional image stack was converted into a three-dimensional mask after background subtraction, using ImageJ/Fiji. Using regions of interest of the head, first and second segment, and third segment and pygidium, the signal in the selected areas was extracted. Due to sample movement during imaging, the signal for one sample from 3 dpf (no food) and one from 3 dpf (*T. suecica*) could not be extracted.

#### 2.7.2. Manual detection of EdU-labelled cells

EdU-labelled cells in a 3 dpf old larva were manually segmented in Napari (RRID:SCR_022765) [31]. For this, cells were individually annotated (segmented) by painting the cell volume.

#### 2.7.3. Statistical analysis of larval size

Variation between the biological replicates and normal distribution was tested with the Shapiro–Wilk test and one-way analysis of variance (ANOVA), respectively. H_0_ was rejected for three of 51 replicates, and a parametric test was used subsequently.

Comparison between groups and age has been done with one-way ANOVA, and Holm–Bonferroni correction for multiple comparison analysis. Pairwise analysis was performed with a paired *t*‐test and corrected using Holm–Bonferroni correction.

#### 2.7.4. Statistical analysis of feeding onset and lipid droplets

Fisher’s exact test was used to test for variation between the biological replicates and a significant association between the variables. Holm–Bonferroni correction was performed for multiple comparison analysis.

## 3. Results

### 3.1. Feeding source affects feeding onset, growth and lipid droplet consumption

To determine when the larvae start feeding and whether they have any food preferences, larvae of *P. dumerilii* were exposed to either *T. suecica* or *G. marina* for 12 h (figure 1*c*). For both algae, larvae started feeding between 5 dpf and 5.5 dpf, though significantly more larvae had fed on *G. marina* by the end of the 12 h feeding period (figure 1*d*,*h*–*j*).

Next, I assessed the growth rate and lipid droplet depletion to evaluate the effect of food on larval growth and the consumption of maternally provided resources. For this experiment, 1.5 dpf larvae were continuously raised with either *T. suecica* or *G. marina*, and their size and absence of lipid droplets were recorded (figure 1*e*,*g*). Before feeding onset, the unfed and fed larvae grew at the same rate. A potential explanation for different growth rates could have been that secreted chemicals from the algae are sensed, inducing growth and altering the developmental trajectory, even before feeding onset.

After 7 dpf, unfed larvae grew significantly slower than fed larvae, despite still having access to the maternally provided lipid droplets (figure 1*g*). After 7 dpf, the unfed larvae appeared to consume the lipid droplets at a slower rate than fed larvae, though this effect was not significant (electronic supplementary material, S2).

### 3.2. Increased cell proliferation in head and posterior growth zone

*Platynereis dumerilii* grows continuously throughout its life by adding new segments at the posterior growth zone. To investigate the number and location of new cells, I performed a cell proliferation assay for 6 h each day in 3–8 dpf larvae (figure 1*b*).

Differences in growth rate between fed and unfed larvae are reflected in the amount and timing of cell proliferation. Until 5 dpf, unfed and fed larvae develop at a similar rate, with exceptionally high cell proliferation rate in the 3 dpf larvae (figure 1*e*,*f*). At this stage, the larva consists of over 9000 cells [32]. By manually segmenting the EdU-labelled cells in one sample, I found that between 72 hpf (hours post fertilization) and 78 hpf, more than 800 new cells developed (electronic supplementary material, video S1).

**Figure 2.**
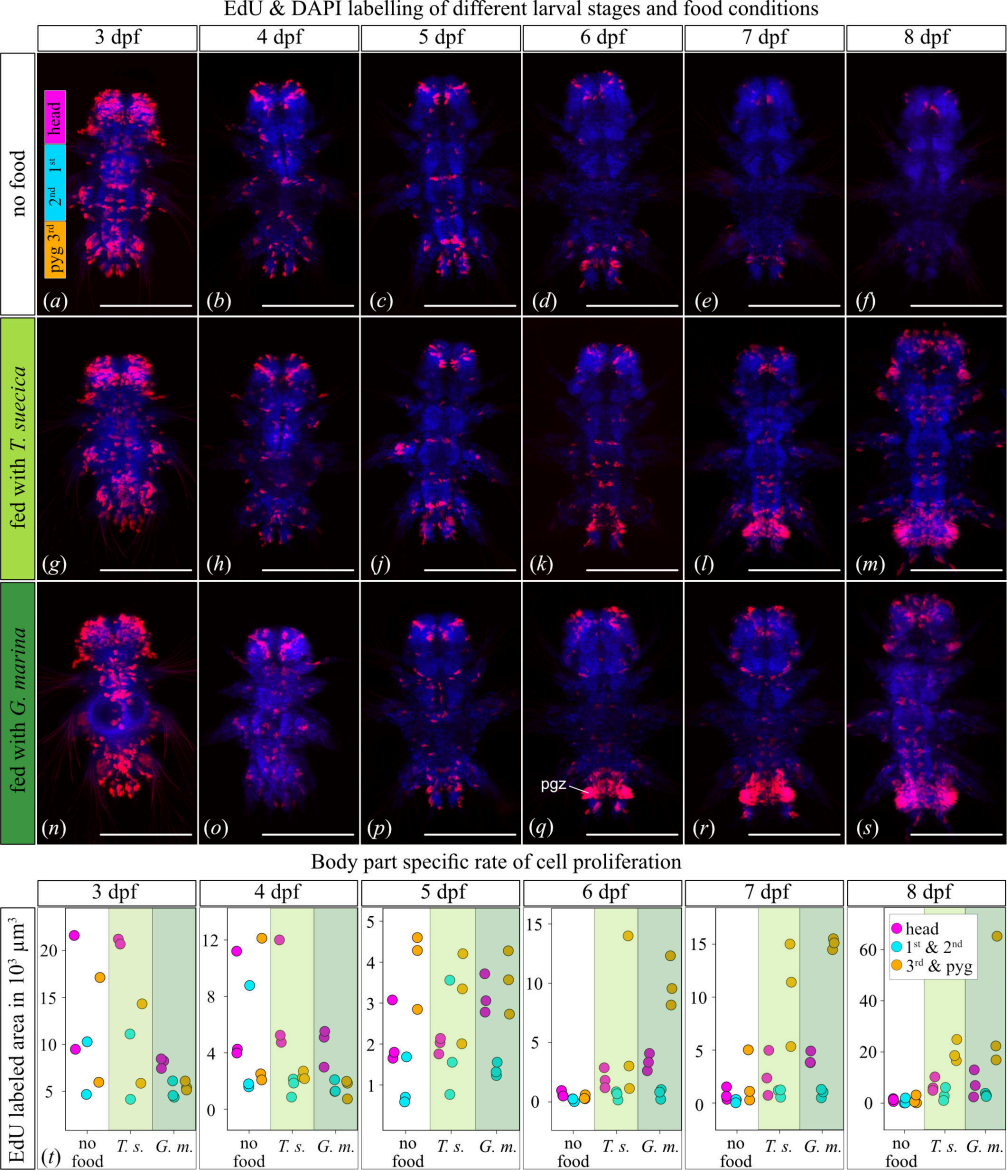
Cell proliferation across larval stages and food conditions. (*a–s*) Fluorescence micrograph of cell proliferation (red) counterstained with DAPI (blue). The labelled regions in (*a*) correspond to (*t*) where cell proliferation is estimated in the head, first and second segments, and third segment and pygidium. Three larvae aged three to eight days were analysed for different food conditions. pgz, posterior growth zone; G. m., *G. marina*; T. s., *T. suecica*. Scale bar, 100 µm.

However, in larvae older than 5 dpf, cell proliferation significantly differs between fed and unfed larvae. Unfed *P. dumerilii* further reduced cell proliferation throughout the body, whereas fed larvae showed increased cell division (figure 1*f*). The slightly earlier onset of cell proliferation in the posterior growth zone in 6 dpf old *G. marina*-fed larvae further supports the observed trend that larvae fed with the diatom grow initially faster than those fed with *T. suecica* (figures 1f,2 and 2*k*,*q*). In addition, fed larvae had weak cell proliferation in the trunk segments but a noticeable increase in newly developed cells in the head, likely contributing to brain development (figure 2).

## 4. Discussion

In this study, I investigated how different food types affect late larval development in *P. dumerilii*. The larvae can settle between 3 and 4 days post fertilization in response to the settlement cue, *G. marina* [29]. However, in the absence of *G. marina* or other food sources, such as *T. suecica*, the competent larvae delay settlement.

### 4.1. Food availability affects larval development

This work demonstrates how competent *P. dumerilii* larvae respond to the lack of food by pausing cell proliferation throughout the whole body. These results complement a previous study showing that the larvae can survive for up to 30 days without food, remaining at the three-segment stage [33]. Nutritional modulation of development is not unique to *P. dumerilii* and has been described in many other taxa, in which insufficient nutrients impact developmental trajectories, alter cell metabolism and influence resource allocation during organogenesis [34].

Before the onset of feeding, food availability does not significantly impact growth rate and cell proliferation in *P. dumerilii*. However, once larvae begin feeding, cell proliferation is initiated in the head and posterior growth zone. In addition, the data suggest that feeding larvae may deplete the maternally provided lipid droplets faster than starved ones.

A possible explanation for these differences in resource utilization and development is that starved larvae limit energy consumption to prolong their planktonic phase, thereby increasing the time available to them for finding a suitable habitat before depleting energy resources. In contrast, feeding larvae maximize resource use of food and lipid droplets to increase their body size, which may enhance their ability to compete with conspecifics for food and space.

### 4.2. Feeding onset depends on food type

My data show that *Platynereis* begins feeding between 5 and 5.5 dpf, nearly two days after reaching competency for settlement. The slightly earlier feeding onset with *G. marina* may be due to greater accessibility of the biofilm, compared with the free-swimming *T. suecica*. Alternatively, since *G. marina* induces earlier settlement, it may also accelerate larval development, enabling earlier food uptake, though evidence remains lacking [29].

### 4.3. Summary and outlook

This work paves the way for future studies on the molecular mechanisms underlying the nutritional modulation of development. Due to its small size and life history, *Platynereis* provides a valuable model for investigating the crosstalk between nutrition, metabolism and development to gain a detailed mechanistic understanding of resource allocation, storage and the effect of maternally provided yolk on early development.

## Data Availability

Python 3.7 scripts and ImageJ Macro are available at commit nrandel_statistics_d64cdf3_2025-04-29 (29 Apr 2025) on GitHub [35]. The datasets supporting this article have been uploaded as part of the electronic supplementary material [36].
